# Longitudinal Study of *Mycobacterium avium* Subsp. *paratuberculosis* Antibody Kinetics in Dairy Cattle Using Sera and Milk throughout the Lactation Period

**DOI:** 10.3390/vetsci7030081

**Published:** 2020-06-30

**Authors:** Md. Shohel Al Faruk, Young-hoon Jung, Tai-young Hur, Sang-suk Lee, Yong-il Cho

**Affiliations:** 1 Department of Animal Science and Technology, Sunchon National University, Suncheon, Jeonnam 57922, Korea; 1175059@s.scnu.ac.kr (M.S.A.F.); rumen@scnu.ac.kr (S.-s.L.); 2 National Institute of Animal Science, Cheonan, Chungnam 3100, Korea; vet9973@korea.kr (Y.-h.J.); tyohur@korea.kr (T.-y.H.)

**Keywords:** antibody kinetic, ELISA, paratuberculosis

## Abstract

*Mycobacteriumavium* subsp. *paratuberculosis* (MAP) is the causative agent of Johne’s disease in dairy cattle populations around the world. The objective of this study was to evaluate MAP antibody kinetics in serum and milk samples throughout the lactation period in dairy cattle. The samples were collected simultaneously from eight MAP-positive and two healthy MAP-negative (control group) cows. The MAP antibody was detected by using serum and milk ELISA. The serum and milk MAP antibody titers fluctuated between the positive and negative cut-off values in this study. Specifically, cattle with low MAP antibody titer (<100) showed fluctuation between the cut-off values. Variable changes of MAP antibody titer were also observed after parturition. Between the serum and milk MAP antibody titers, there was a positive correlation (R^2^ = 0.5358) observed throughout the assessment period. The milk MAP ELISA test had low diagnostic performance in cows with low MAP titer due to its weak correlation (R^2^ = 0.0198). Finally, this study suggest that the periodic MAP ELISA test is recommended for the application of Johne’s eradication program due to the fluctuating nature of MAP antibody kinetics.

## 1. Introduction

Paratuberculosis, commonly known as Johne’s disease, is a production-limiting disease of dairy cattle caused by *Mycobacterium avium* subsp. *paratuberculosis* (MAP) and has a substantial financial effect on the dairy industry [1]. The disease was first reported in Korea in 1984 and its prevalence was subsequently reported to be 18.7% and 11.7% in dairy and beef cattle, respectively [2]. In Korea, prevalences of 6.1% and 1.2% were recorded in dairy and beef cattle, respectively, in Gyeongnam province in 2009 [3], and a 5.2% prevalence was observed in Jeju province in 2013 [4]. Previous studies have reported outbreaks of MAP in other countries. A cattle herd MAP prevalence of 16.7% was reported in Canada, and a 68% MAP prevalence was recorded in US dairy farms in 2008 [5]. Johne’s disease results in decreased milk production and increased cow replacement costs, which has resulted in economic losses in the US dairy industry estimated at US$ 200 to US$ 250 million annually, or US$ 22 to US$ 27 per cow [6].

The main clinical signs of paratuberculosis are persistent diarrhea, reduced milk production, weight loss, and progressive emaciation. This infectious disease develops slowly and is characterized by chronic degenerative granulomatous enteritis that affects the distal part of the small intestine, as well as the colon and associated lymphoid tissue. Young animals have a greater possibility of getting an MAP infection when their age is below six months. Following infection by MAP, seroconversion usually occurs around two years of age [7]. Not all infected animals develop clinical signs, but the disease can be detected by diagnostic testing a few years after the initial infection [8].

Therefore, adequate diagnostics are essential to reduce the prevalence of MAP, which is a key component of the control of paratuberculosis in a farm [9]. Fecal culture and PCR are the diagnostic tools for detection of the MAP antigen, whereas the ELISA methods are commonly used for detection of MAP antibodies. However, fecal samples sometimes show negative results in true MAP-positive animals due to the intermittent shedding of bacteria, resulting in differing sensitivity of each test depending on the stage of infection [10,11]. Although the serum/milk ELISA has low sensitivity and high specificity, it is commonly used in dairy herds for detection of MAP, due to its acceptable diagnostic performance, as well its time efficiency and cost effectiveness.

Dairy farms participating in the Johne’s disease eradication program in Korea perform MAP screening tests by using antibody detection ELISA regularly, once or twice per year, with irregular fecal antigen detection PCR, and removing MAP-positive animals from the herd. However, annually new MAP-positive animals have been coming out of these participating farms for several years. We presumed that MAP-positive animals were being missed by single time screening, indicating that the evaluation of MAP antibody status is needed throughout the animal’s lifetime. Therefore, the objective of this study was to evaluate MAP antibody kinetics in serum and milk samples throughout the lactation period and to assess the diagnostic performance of the milk ELISA test.

## 2. Materials and Methods

### 2.1. Ethical Statement

All animal procedures and the study design were approved by the Institutional Animal Care and Use Committee (IACUC) at the National Institute of Animal Science (NIAS), Republic of Korea. The Reference number was NIAS (2013-046).

### 2.2. Selection of Animals

A longitudinal study was performed on three commercial dairy farms that were enrolled in a Johne’s disease control program in Korea. The cows on these farms underwent one- or two-time screenings per month for Johne’s disease by using MAP antibody detection ELISA (IDEXX Laboratories, Inc. Westbrook, ME, USA). More than a hundred cows were tested from three dairy farms and among them eight cows were diagnosed as MAP-positive during the screening period. The cows had no apparent clinical signs of Johne’s disease and showed normal reproductive and milk yield performance. Two healthy MAP-negative dairy cows were used as a negative control. The control group animals were negative in both fecal PCR and serum/milk ELISA results. Cow-specific information about age, calving date, and milk production was recorded for all tested cows. The ages of all animals ranged from 4 to 8 years. The animals were housed in free-stall housing systems and their average daily production of milk was 27–36 kg/cow.

### 2.3. Sampling and MAP Antibody Testing

Blood and milk samples were collected simultaneously for MAP antibody ELISA testing from eight MAP-positive and two healthy MAP-negative cows throughout the year. We collected blood and milk samples one to two times per month; however, a few samples were missed due to inappropriate sample handling or recording. Only blood samples were collected during the dry period. Approximately 5 mL of blood was collected from the jugular vein of each animal and transferred to the laboratory for further processing. For serum preparation, blood samples were centrifuged at 1800× *g* for 15 min. The serum was then transferred into a screw-cap tube and stored at −20 °C until analysis. Serum samples were tested by MAP-specific antibody ELISA (IDEXX Laboratories, Inc. Westbrook, ME, USA), following the manufacturer’s recommendations. Samples with a sample-to-positive (S/P) ratio of 55 or higher were considered seropositive for MAP antibody detection, as determined by IDEXX kit cutoffs.

Milk samples were collected aseptically from all four teats after discarding the first few streaks. The milk was taken in a vial, transported to the laboratory, and stored at 4 °C until processed. Samples were tested using a commercially available MAP antibody detection ELISA (IDEXX Laboratories, Inc. Westbrook, ME, USA) as per the procedure recommended by the manufacturer. To determine the number of animals that were MAP-antibody-positive by milk ELISA, an S/P ratio of 30 was used as the cut-off value for a MAP-positive test result, as recommended by IDEXX.

### 2.4. Statistical Analysis

Serum and milk ELISA S/P ratios were compared by using correlation analysis and controlling for repeated measures. Pearson’s correlation coefficient (R^2^) was calculated to determine the linear relationship between test results, and the differences were considered significant at *p* < 0.05. Interpretation of the R^2^ values was done as previously reported [12,13,14]; that is, strong agreement (1 > R^2^ > 0.7), moderate agreement (0.7 > R^2^ > 0.5), weak agreement (0.5 > R^2^ > 0.3), and none or very weak agreement (0.3 > R^2^ > 0).

## 3. Results

**Figure 1:**
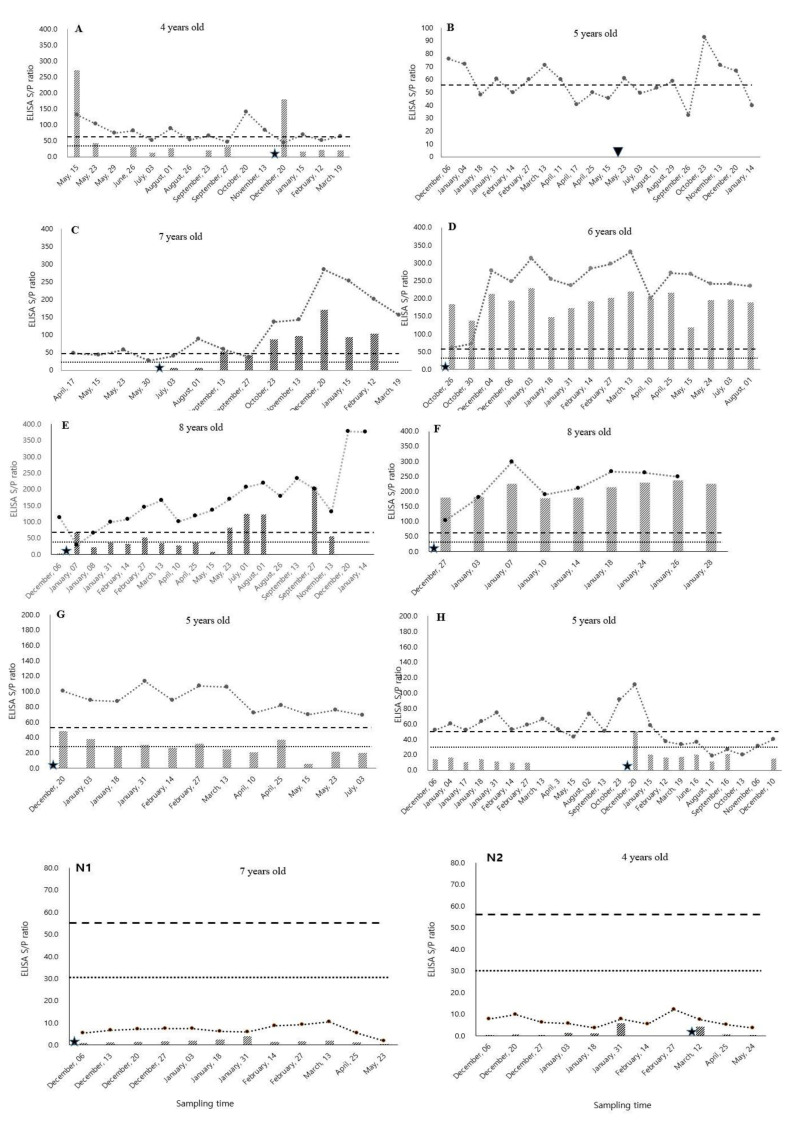
Longitudinal temporal changes of *Mycobacterium avium* subsp. *paratuberculosis* (MAP) in the outcome of milk and serum ELISA in MAP-positive cows (cows **A** to **H**) and in control group (Cows **N1** and **N2**). Each measurement reflects a test-day sampling result. Line graph: sample-to-positive ratio (S/P) of serum ELISA; bar diagram: S/P of milk ELISA; striped line: cut-off value for serum ELISA (S/P = 55); dotted line: cut-off value for milk ELISA (S/P = 30). The symbols ★ and ▼ indicate time of calving and abortion, respectively. Month and date reflect sampling time.

The eight MAP positive cows (denoted as cows A to H) were enrolled as Johne’s disease cows and their MAP antibody levels were observed in serum and milk ELISA throughout the year (Figure 1). Two healthy MAP-negative cows were used as negative control animals (cows N1 and N2) and their MAP ELISA testing was performed for six months (Figure 1). All animals had different age records and normal calving history, except one animal that had a history of abortion (cow B).

### 3.1. MAP Antibody Fluctuation

The antibody level of all MAP-positive animals fluctuated throughout the experimental period. (Figure 1). In cow A, the serum and milk MAP antibody titers fluctuated between the cut-off values. The serum MAP antibody titers were above the cut-off level in the first to fourth observations, then it decreased and again increased for a single-time observation and continued to maintain its fluctuating nature. The serum MAP antibody titer of cow A was above the cut-off level at ten different times throughout the study period and was below the cut-off level at five other times. Most of the serum MAP antibody levels were higher than the milk MAP antibody levels over the observational period; although, at two observations, the milk MAP antibody titer was higher than the serum titer. Cow B aborted twin calves in the fourth month of pregnancy, and its serum MAP antibody titer fluctuated within the borderline of the cut-off value over the study period. The serum MAP antibody titer of cow H also fluctuated between the cut-off points followed by decreased antibody titer after calving. In general, the MAP antibody titer of cows A, B, and H fluctuated between the borderline of cut-off value. Due to the fluctuating nature of the MAP antibody and depending on the sampling time, these MAP-positive animals would be diagnosed as MAP-negative.

### 3.2. Dynamics of MAP Antibody Changes After Parturition

*Increased trend*: The changing pattern of MAP antibody kinetics in the serum and milk samples (cows A to H) observed after parturition (Figure 1). The antibody titer of cows C, D, E, and F showed an increased trend in MAP titer after parturition. Based on our field experience, we also categorized our animals into high (>200) and low (<100) MAP antibody titer groups. The serum and milk MAP antibody kinetics of cows C and E fluctuated at a low titer level (<100) until mid-lactation and then the fluctuation continued at a high titer level (>200) throughout the remaining period. The serum MAP antibody kinetic pattern did not follow the milk MAP antibody kinetic pattern before mid-lactation, but afterward, both the serum and milk MAP kinetics followed similar patterns.

Cows D and F showed a rapidly increasing trend of MAP antibody titers after parturition, and their serum MAP antibody titer was higher than that of milk except for one or two unusual observations. The patterns of MAP antibody changes were similar in serum and milk over the entire observational period, with the antibody titers being high in both serum and milk. Cow F had clinical symptoms of Johne’s disease, including watery diarrhea, weight loss, and dehydration, after its fourth calving, and samples of its serum and milk were collected more frequently for one month after it developed clinical symptoms. In cow F, its milk MAP antibody titers were approximately 85% of the serum levels.

*Steady-state/decreased trend:* After parturition, the MAP antibody kinetics of cow G maintained a steady-state trend in MAP antibody titers, whereas cows A, B, and H showed a decreasing trend. In the case of cow G, the serum MAP antibody levels were higher than those in milk, and both the serum and milk MAP antibody titers maintained a steady-state pattern after parturition. The milk MAP antibody titer remained near the cut-off value over the entire study period. The MAP antibody titers of serum and milk for cow G were 100.5 and 48.4, respectively, at the first observation after calving, both of which were higher than the standard cut-off values. The MAP antibody titers of cow A showed a decreased trend and maintained their fluctuating nature after parturition. Cow B also showed a decreasing and fluctuating trend after abortion. In cow H, the serum MAP antibody titer fluctuated until parturition and then sharply increased followed by a gradual decrease to below the cut-off value at one month after parturition.

For a comparison of MAP-negative and MAP-positive cows, two healthy cows (N1 and N2) were selected as a MAP-negative controls and their MAP antibody levels in both serum and milk were observed throughout the observational period (Figure 1). Cows N1 and N2 maintained low S/P ratios due to the non-specific reaction of the ELISA test kits. The average ELISA S/P ratios were <10 in serum samples and <5 in milk samples in both MAP-negative animals.

### 3.3. Correlation between Serum and Milk MAP Antibodies

**Figure 2:**
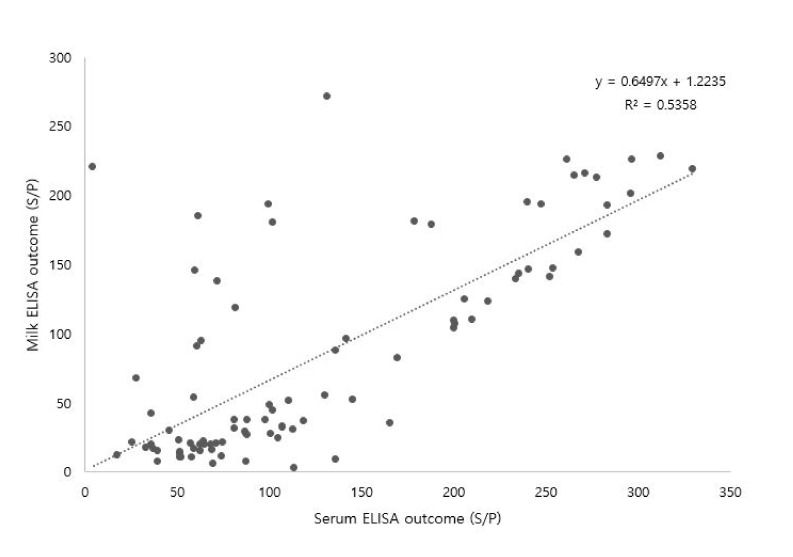
The correlation between the outcomes of serum and milk ELISA results (180 measurements; 7 cows; R^2^ = 0.5358; *p* ≤ 0.00). The cut-off value used for the milk samples depends on a sample-to-positive (S/P) ratio ≥ 30 while that of the serum samples was S/P ≥ 55. Each dot indicates one milk and serum ELISA outcome (cows were measured repeatedly).

**Figure 3:**
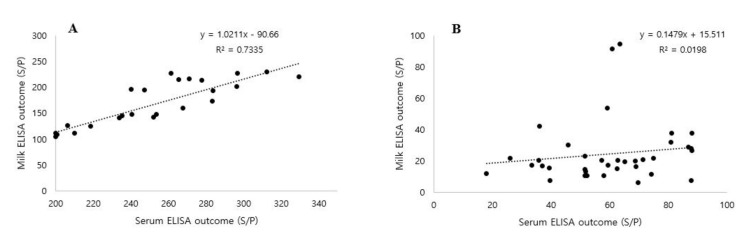
The correlation between the outcome of serum and milk ELISA results in high and low serum MAP antibody titer. (**A**) High serum MAP antibody titer (>200) with an R^2^ of 0.7335 (*p* ≤ 0.00). (**B**) Low serum MAP antibody titer (<100) with an R^2^ of 0.0198 (*p* ≤ 0.00). Each dot reflects 1 milk and serum result.

The repeated serum and milk ELISA measurements underwent correlation-based analysis (Figure 2 and Figure 3). Approximately 180 serum and milk samples were collected from the MAP-positive cows and analyzed by ELISA. The serum and milk ELISA results showed a moderate agreement (R^2^ = 0.5358) between the serum and milk MAP antibody levels of the MAP-positive cows (Figure 2). For further analysis, the MAP antibody titers were categorized as high (>200) or low (<100) level (Figure 3). A strong agreement (R^2^ = 0.7335) was observed for the relationship between the serum and milk sample titers in the high MAP antibody titer group (>200), which indicates that serum and milk MAP antibody titer patterns were similar (Figure 3A). However, the low MAP antibody titer group (<100) showed a very weak correlation between serum and milk samples (R^2^ = 0.0198), indicating that the milk MAP antibody pattern did not closely follow the serum MAP antibody pattern (Figure 3B), indicating that the milk ELISA test would provide low diagnostic performance in low MAP antibody titer cows.

## 4. Discussion

### 4.1. Fluctuation of MAP Antibody Titers

The kinetics of cattle serum and milk MAP antibody titers in MAP-positive cows were compared over a year in this longitudinal study. Repeated sampling within the study period helped to make unique MAP antibody profiles in serum and milk of individual cows throughout the lactation. In particular, the relationships between MAP antibody levels in serum and milk were examined, with the results revealing the fluctuating nature of MAP antibody levels in both serum and milk (Figure 1). The MAP antibody level fluctuated between the cut-off value in those cattle (cows A, B, and H) that had low antibody titer (<100). The serum MAP antibody titer of cows A, B and H fluctuated in between the border line of the cut-off value. This fluctuating of low antibody titers might be associated with transient paratuberculosis infections in dairy cattle. Transient MAP shedding due to the uptake of MAP from the environment has been previously described [15], and that transience may lead to a transient antibody response. A low antibody titer is also associated with the initial stages of MAP infection, during which the cow’s immune system can control the MAP infection, resulting in low titer observed. Cow B was a more typical example of MAP antibody fluctuations, in which the serum MAP antibody titer is negative for approximately half of the MAP tests over the study period. Although a previous study also showed MAP antibody fluctuation over the period [16], our results reveal that the fluctuation was observed particularly in between the cut-off value. This fluctuation pattern indicates the possibility of missing true MAP-positive animals by relying on a single-point observation. Additionally, if the MAP antibody titer lies near to the cut-off boundary, those cow should be listed as potentially MAP-positive and should undergo more frequent testing to avoid false MAP-negative results in farms that have launched a Johne’s eradication program.

### 4.2. Increasing Trend of MAP Antibody Titers after Parturition

Variable changes in MAP antibody titers were also observed after parturition (Figure 1). A trend toward an increase MAP antibody level (cows C, D, E, and F) was observed after calving in this study. In calving animals, such an increase has been associated with physiological stress and a depressed immune system [17]. Cows C and E exhibited gradually increased trends with high MAP antibody titers. The low MAP antibody titers were present until the mid-lactation period, after which their titers were notably increased. This pattern change might be due to the cows being in different stages of MAP infection. In the early stage of MAP infection, the cow’s immune system can control the infection resulting in the low MAP titer observed, whereas the MAP antibody titer is markedly increased due to immune suppression during the longer lactation period [18]. Cows D and F had sharply increased trends, with the higher MAP antibody titer observed after parturition, which might be associated with the immune suppression related to calving and milking stress during the periparturient period [11]. The bacterial load during infection is commonly high when immune systems are depressed, resulting in the high MAP antibody titers observed in cows D and F. Cow F showed clinical signs of Johne’s disease along with its high MAP antibody titer. The increase to a high MAP antibody titer in cow F is indicative of a high possibility of the cow developing clinical signs of MAP [19].

### 4.3. Steady or Decreasing Trend of MAP Antibody after Parturition

A trend to a steady-state or decreasing MAP antibody (cows A, B, G, and H) was observed after parturition, followed by a fluctuating nature, in the study. This might be due to the presence of transient MAP infections in these cattle, as previously reported in dairy cattle [7]. In addition, during the early stage of Johne’s disease, the MAP antibody titer can fluctuate because of the immune system of the cow restricting the development of MAP infection [20,21]. Although the serum MAP antibody titer was continuously positive after parturition, most of the milk MAP ELISA results were negative (i.e., less than the cut-off value). This may be due to the dilution of MAP antibodies in milk to a level below the detection limit, as has been previously suggested [22,23]. Moreover, low titer animals had higher milk production than those of high titer animals, and the increased milk production may lead to milk ELISA underestimating MAP antibody level [24,25]. Another study suggested that for every 5 kg increase in milk production, the S/P ratio should be multiplied by 0.89. Moreover, during the early stages of Johne’s disease, milk MAP antibody detection can be affected by the dilution effect of increased milk production [22,26].

### 4.4. Effect of Age on MAP Antibody Titer

The MAP antibody titers were influenced by the age of the cows. Cows that were four to five years old had lower antibody titers compared to older cows, which is in agreement with previous reports [24]. Although the sample number was limited in the study, the results show that the MAP antibody titer increases with animal age. Generally, calves become infected by MAP during their first year of life, and paratuberculosis is known to have a long subclinical phase in which there are undetectable amounts of MAP-specific antibodies present [27]. This general pattern of paratuberculosis progression with age has also been indicated by the observation of an increased S/P ratio in ELISA of MAP-positive cows tested repeatedly over time [24].

### 4.5. Correlation between Serum and Milk MAP Antibodies

The relationship between the serum and milk MAP ELISA results was significant in this study (Figure 2). The 180 serum and milk samples showed a moderate-level positive correlation (R^2^ = 0.5358, *p* ≤ 0.00) between serum and milk MAP antibody levels. These results are in agreement with Li et al. where a positive correlation was also observed between serum and milk MAP antibodies and the R^2^ value ranged from 0.572 to 0756 [28]. In addition, a high MAP antibody level in blood was accompanied by increased detection of MAP in the milk of clinically affected cows [29,30]. For more clarification of this relationship, we categorized the data into high (>200) or low (<100) MAP antibody titer. Strong agreement was observed between serum and milk when the antibody titer was high (>200). A high antibody titer is produced during the terminal stage of Johne’s disease, resulting in depression of the immune function and cows are being unable to control the disease [31]. Whereas in animals with a low (<100) MAP antibody titer, a weak agreement was observed in this study (Figure 3) because this condition typically occurs only during the initial stages of the disease, when the immune system of the cow is able to control the bacterial infection. Serum and milk ELISA showed similar results when the MAP antibody titer was above 200. Heavily infected animals carry high levels of antibodies in both serum and milk. In such cases, milk ELISA would be an effective tool for the diagnosis of MAP in a herd. However, if the serum MAP antibody titer is below 100, the milk MAP ELISA is unable to detect a MAP infection due to the low specificity of the milk test. This result indicates that the milk MAP ELISA has low diagnostic performance in the detection of an early stage MAP infection as the obtained low MAP antibody titers may be below the cut-off value. The milk MAP ELISA test might be useful for general MAP screening in a herd, but for an applications such as a MAP eradication program, serum ELISA should be used due to its higher diagnostic performance compared to that of milk ELISA.

Our study has some limitations. The sample number of MAP positive cows was comparatively low, although more than hundred cows were tested for primary screening. A higher number of MAP-positive cows should be included to determine the certainty of our results. However, the strength of this study is that both serum and milk samples were collected simultaneously from both MAP-positive and MAP-negative control animals throughout the year to observe MAP antibody kinetics. To the best of our knowledge, this is the first recently conducted study in which simultaneous serum and milk samples were obtained over a year to observe the longitudinal changes in MAP antibody level in dairy cattle.

## 5. Conclusions

MAP antibody titers fluctuated in both serum and milk samples over the year, with the fluctuations occurring near the MAP-positive and MAP-negative cut-off borderline. These fluctuations make it difficult to diagnose a MAP-positive cow by only a single time measurement. The study result indicates that periodic MAP screening in a dairy herd is needed due to the fluctuating trend in MAP antibody level. In addition, the serum MAP antibody levels were gradually increased in high MAP antibody titer (>200) cows after parturition. However, some cows showed steady-state or decreasing trends in MAP antibody levels in low titer (<100) cows after parturition. There was a significant relationship between serum and milk sample results in cows with high MAP antibody titer (>200), but a weak relationship in cows with low (<100) MAP antibody titer. This weak agreement between serum and milk samples in low MAP antibody titer cows is indicative of low diagnostic performance of the milk MAP ELISA. Finally, the results of this study suggest that those farms applying Johne’s disease eradication programs should list cows as potentially MAP positive if their antibody titer lies near the cut-off value and periodic MAP ELISA testing is recommended due to the fluctuating nature of MAP antibody kinetics in dairy cattle.
